# Evaluation of the long-term patient-reported outcomes after hip arthroplasty or joint preserving with Sugioka femoral osteotomy in patients with femoral head osteonecrosis

**DOI:** 10.1007/s00264-024-06118-3

**Published:** 2024-02-20

**Authors:** Takeshi Shoji, Hideki Shozen, Shinichi Ueki, Hiroki Kaneta, Yuji Yaunaga, Nobuo Adachi

**Affiliations:** 1 Department of Artificial Joints and Biomaterials, Graduate School of Biomedical Sciences, Hiroshima University, 1-2-3 Kasumi, Minami-Ku, Hiroshima, 734-8551 Japan; 2 Department of Orthopaedic Surgery, Graduate School of Biomedical Sciences, Hiroshima University, 1-2-3 Kasumi, Minami-Ku, Hiroshima, 734-8551 Japan; 3 Department of Orthopaedic Surgery, Hiroshima Prefectural Rehabilitation Center, 295-3 Taguchi, Saijo-Town, Higashi-Hiroshima, 739-0036 Japan

**Keywords:** Patient-reported outcome measures, Osteonecrosis of the femoral head, Osteotomy, Total hip arthroplasty

## Abstract

**Purpose:**

This study retrospectively evaluated long-term clinical outcomes and patient-reported outcome measures (PROMs) in patients with osteonecrosis of the femoral head (ONFH) who underwent transtrochanteric rotational osteotomy (TRO), curved varus osteotomy (CVO), and total hip arthroplasty (THA).

**Methods:**

We retrospectively reviewed the 109 hips in 96 patients (46 men, 50 women) who underwent CVO, TRO, or THA for ONFH treatment. The mean follow-up period for the TRO, CVO, and THA groups was 14.8, 11.5, and 13.3 years, respectively.

**Results:**

The THA conversion rate of the TRO patients was significantly higher than that of the patients with CVO, and the final clinical scores in the patients with TRO did not improve compared with preoperative scores. Postoperative PROMs showed that the total and pain scores of the patients with THA were significantly higher than those of patients with TRO and CVO, while the PROM score did not change between patients with TRO and CVO. The analysis further showed that the preoperative type C2, stage 3A, or postoperative type C1 and C2 were significant predictors of decreased final PROM scores.

**Conclusion:**

This study found that CVO and THA are clinically effective treatments for ONFH, with significant improvements compared with preoperative scores. However, THA was associated with significantly higher PROMs and pain scores than those of CVO and TRO in long-term follow-up. Furthermore, our results suggest that postoperative PROMs depend mainly on the preoperative level of collapse and postoperative transposed intact ratio of the articular surface of the femoral head.

## Introduction

Osteonecrosis of the femoral head (ONFH), a serious disease encountered in younger patients, is a devastating condition with multifactorial aetiology. If left untreated, femoral head collapse often occurs and progresses to secondary osteoarthritis (OA) [1]. Joint-preserving procedures have been developed for preserving the femoral head to avoid or delay the need for joint replacement surgery and play an important role in the management of these patients [2–4]

Several types of osteotomies are performed before and after the collapse of the femoral head to transfer pressure from the affected subchondral area of necrosis to the unaffected joint surface [5–7]. Proximal femoral osteotomies, such as transtrochanteric curved varus osteotomy (CVO) [5] and rotational osteotomy (TRO) [6, 7], aim to move the area of necrosis out of the weight-bearing region, which leads to delayed progression or even healing of the necrosis in the treatment of ONFH. Moreover, these techniques may be effective in decreasing intramedullary pressure and preserving blood flow in the femoral head [8]. With the TRO procedure, the necrotic zone of the head-neck fragment is rotated anteriorly or posteriorly around the neck axis to unload the necrotic zone, whereas the necrotic lesion is shifted medially and laterally in the CVO procedure, which typically enables the non-necrotic part of the femoral head to be displaced to the area of weight loading. The success rates of CVO have been reported to range from 90 to 97.3% [9, 10], whereas the success rates of TRO have been reported to be inconsistent, ranging from 17 to 100% [11, 12]. While joint-preserving procedures have shown significantly improved outcomes and should be emphasized for young patients, total hip arthroplasty (THA) remains the most common technique for patients with ONFH, especially for those with collapsed femoral head, despite the poor long-term outcome of THA in younger patients with ONFH [13–15]. Choosing an appropriate treatment is difficult, and there is no consensus on the optimal treatment for patients with ONFH.

Patient-reported outcome measures (PROMs) are important metrics for evaluating the therapeutic effects of possible discrepancies between surgeons and patients with respect to clinical evaluations (in determining a procedure’s efficacy or appropriateness) or healthcare systems (in the context of value-based healthcare). These evaluations are considered useful in the decision-making process regarding joint preservation. Due to the paucity of PROMs in large cohorts with several surgeries for patients with ONFH, there is limited information available on PROMs after osteotomies and THA procedures. In this study, we mainly focused on the long-term results of PROMs after CVO, TRO, and THA surgery and aimed to evaluate the impact of these parameters on postoperative PROMs in patients with ONFH.

## Materials and methods

### Study population

**Fig. 1:**
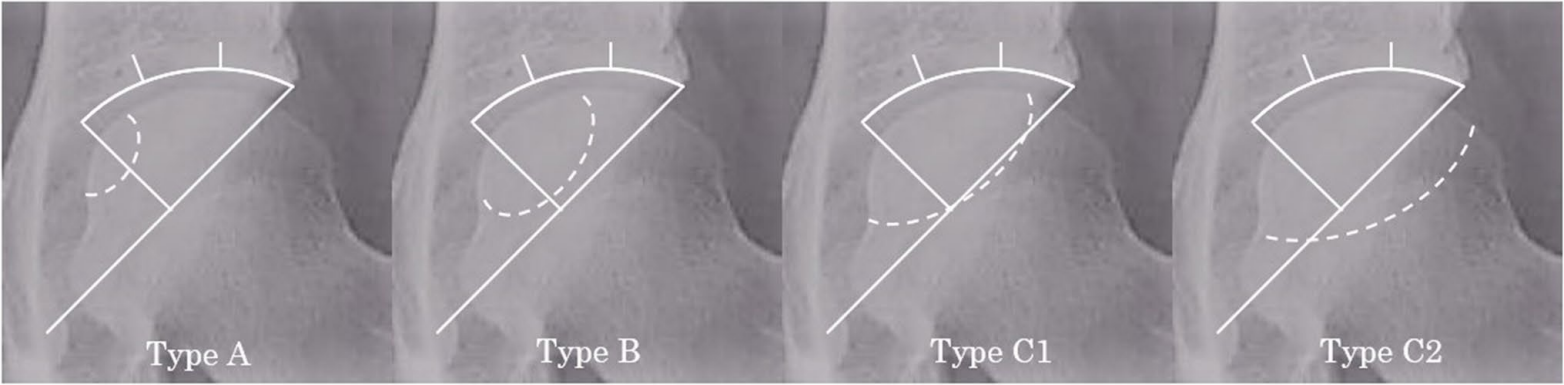
The classification according to the Japanese Investigation Committee of Health and Welfare. Type A indicates that the necrotic area occupies the medial one-third or less of the weight-bearing area. Type B indicates the medial two-thirds or less. Type C1 indicates more than two-thirds but not extending to the acetabular rim. Type C2 indicates more than two-thirds and extending to the acetabular rim

We retrospectively reviewed 109 hip records in 96 patients (46 men and 50 women) who underwent TRO, CVO, and THA for the treatment of ONFH (Table 1). All patients exhibited radiographic evidence of ONFH and the patients who have an osteotomy and THA were excluded in this study. The subset of patients with the localization of the necrotic lesion of type C1 and C2 (Fig. 1) and initial stages of ONFH of stage 2 (indicating radiographically abnormal without collapse), stage 3A (collapse of the femoral head of < 3 mm without OA change), stage 3B (collapse of the femoral head of ≥ 3 mm without OA change), and stage 4 (OA change) according to the Japanese Investigation Committee (JIC) of Health and Welfare classification were included [16]. The patients’ age, sex, body mass index, previous alcohol intake, previous corticosteroid use, preoperative type, and stage of ONFH were recorded. At the final follow-up, the postoperative type and stage of ONFH, Japanese Orthopaedic Association (JOA) score, and Japanese Orthopaedic Association Hip Disease Evaluation Questionnaire (JHEQ) score (0–88 points, where 0 is worst and 88 is best; pain, 0–28 points; movement, 0–28 points; mental, 0–28 points) [17, 18] were also recorded. Postoperative complications requiring revision surgery after osteotomy were identified by reviewing the medical records and serial radiographs. The mean follow-up period was 14.8 years (range, 10.1–26 years) in the TRO, 11.5 years (range, 10.1–12.2 years) in the CVO, and 13.3 years (range, 10–20.0 years) in the THA groups.

**Table 1:** Baseline characteristics of patients

		TRO	VO	THA
Patient	(*n*)	29	33	34
Hip	(hip)	31	37	41
Gender	(*n*)	M, 17; F, 12	M, 14; F, 19	M, 15; F, 19
Age	(y.o.)	40.0 (12.7)	38.7 (12.9)	55.3 (11.9)
BMI	(kg/m^2^)	21.9 (2.9)	24.4 (5.2)	23.6 (4.1)
Follow-up duration	(yrs.)	14.8 (4.5)	11.5 (1.4)	13.3 (3.0)
Association				
Steroid	(*n*)	19	23	20
Alcohol	(*n*)	6	5	6
Idiopathic	(*n*)	4	5	8
ONFH type				
Type C-1	(*n*)	10	31	19
Type C-2	(*n*)	21	6	22
ONFH stage				
Stage 2	(*n*)	11	21	5
Stage 3A	(*n*)	16	16	13
Stage 3B	(*n*)	4	0	6
Stage 4	(*n*)	0	0	17

*TRO* transtrochanteric osteotomy, *VO* varus osteotomy, *THA* total hip arthroplasty

Results are expressed as mean (standard deviation)

*M* male, *F* female, *y.o.* years old, *BMI* body mass index, *yrs.* years

*ONFH* osteonecrosis of femoral head

### Indication of the surgery

Osteotomy was performed in patients who preferred hip joint preservation after both osteotomy and THA were explained. To perform osteotomy, in general, the femoral head should have a viable portion of such a size that the restoration of an adequate weight-bearing articular surface is possible after osteotomy. The indications for TRO were < 60 years of age, JIC type C1 or C2, and stage 2, 3A, or 3B ONFH, with one-third or more of the intact area in the posterior region of the femoral head [19]. Furthermore, we indicated the use of CVO in patients who were < 60 years of age, JIC type C1 or C2, and stage 2 or 3A ONFH with sufficient lateral viable bone (> 150° between the central vertical line of the femoral head and the lateral margin of the necrotic portion on a midcoronal MRI scan) [9]. Of the total, 29 patients underwent TRO, 33 patients underwent CVO, and THA was performed on 34 patients.

### Surgical procedure

The surgical technique used for TRO was the same as originally described by Sugioka [6]. The greater trochanter and intertrochanter were osteomized and the femoral head fragment was rotated anteriorly. The degree of anterior rotation ranged from 70 to 90°. During these osteotomies, careful attention was paid to preserving the medial femoral circumflex artery located in the adipose tissue just inferior to the quadratus femoris. CVO was performed according to the technique developed by Nishio and Sugioka [5]. In this procedure, a curved osteotomy was performed between the greater and lesser trochanters. The femoral head was then rotated to the varus position. THA as primary surgery was performed using the posterolateral approach with a cementless prosthesis, and conversion THA was performed using the posterolateral or anterolateral spine approach with a cementless prosthesis.

### Statistical analysis

All data are expressed as mean ± standard deviation (SD) and statistical analysis was performed using statistical visualization software (Stat-View-J, Version 5.0; Hulinks, Tokyo, Japan). The Mann–Whitney *U* test was used to assess differences between the three groups. Statistical significance was set at *p* < 0.05.

## Results

There were no cases of postoperative complications requiring revision surgery, such as infection, non-union, or fracture, in the TRO, CVO, and THA groups. Thirteen (42.0%) patients in the TRO group and four (10.8%) in the CVO group showed postoperative collapse of the femoral head and OA progression that required conversion to THA, which showed a significant difference between the two groups (Table 2). Regarding ONFH stage grade progression, there were six cases with no progression (19.4%), while ten cases showed one-stage progression (32.3%), and 15 cases showed two-stage progression (48.4%) in the TRO group. In contrast, there were 24 cases with no progression (64.9%), which showed a significant difference compared with the TRO group, three cases with one-stage progression (8.1%), and ten cases with two-stage progression (27.0%) in the CVO group. In the THA group, one patient showed postoperative hip dislocation, whereas there were no other complications such as aseptic loosening.

**Table 2:** Post-operative radiographic character

Post-operative	(*n*)	TRO (31)	VO (37)	
ONFH type				
Type B		7	23	
Type C-1		19	14	
Type C-2		6	0	
Stage progression				
No change		6	24	*
1 stage		10	3	
2 stage		15	10	
THA conversion		13	4	*

*ONFH* osteonecrosis of femoral head, *p < 0.05

*TRO* transtrochanteric osteotomy, *VO* varus osteotomy

*THA* total hip arthroplasty, JIC classification

The postoperative JOA score showed a significant improvement in gait and activities of daily living (ADL) scores in the TRO group; total, pain, gait, and ADL scores in the CVO group; and total and all sub-scores in the THA group compared with the preoperative scores. However, the postoperative range of motion (ROM) score decreased compared with the preoperative score with significance in the TRO group (Table 3). The postoperative total and pain scores in the THA group were significantly higher than those in the TRO and CVO groups. However, there were no significant differences in ROM, gait, or ADL scores among the groups.

**Table 3:** Pre- and post-operative JOA score

		Total			Pain			ROM			Gait			ADL		
		Pre-op	Post-op		Pre-op	Post-op		Pre-op	Post-op		Pre-op	Post-op		Pre-op	Post-op	
TRO		63.2 (13.2)	67.9 (17.7)		21.9 (10.3)	22.5 (11.1)		18.0 (4.7)	14.6 (4.1)	*	11.4 (2.7)	14.5 (3.7)	*	11.9 (2.7)	14.1 (2.5)	*
VO		58.2 (15.1)	71.9 (14.9)	*	15.8 (7.9)	24.1 (9.4)	*	17.5 (3.0)	16.8 (2.4)		10.5 (4.7)	13.5 (4.9)	*	13.1 (4.1)	16.4 (2.8)	*
THA		47.5 (8.3)	83.0 (12.6)	*	11.7 (9.9)	33.9 (6.4)	*	14.9 (3.4)	17.0 (2.6)	*	9.7 (4.0)	16.0 (3.9)	*	12.0 (2.0)	15.9 (3.3)	*

*JOA* Japanese Orthopaedics Association, Results are expressed as mean (standard deviation), *p < 0.05

*pre-op.* pre-operative score, *post-op.* post-operative score, *TRO* transtrochanteric osteotomy, *VO* varus osteotomy, *THA* total hip arthroplasty

Regarding the postoperative JHEQ score at the final follow-up, the visual analog scale (VAS) and total pain scores in the THA group were significantly higher than those in the TRO and CVO groups, and the total score in the CVO group was significantly higher than that in the TRO group (Table 4). However, there were no significant differences in movement and mental scores among the three groups, and in VAS and pain scores between the TRO and CVO groups. In ONFH patients with preoperative JIC type C1, there were no significant differences in the JHEQ total and pain scores among the groups, whereas the total and pain scores of the THA group were significantly higher than those of the TRO and CVO groups in patients with preoperative JIC type C2. Similarly, there were no significant differences in the total and pain scores among the groups in patients with preoperative JIC stage 2, whereas the scores of the THA group were significantly higher than those of the TRO and CVO groups in patients with preoperative JIC stage 3A. Moreover, the total and pain scores of the stage 2 patients were significantly better than those of the stage 3 patients in the osteotomy group (*p* < 0.05). Patients with postoperative JIC type B had significantly higher total and pain scores than those with postoperative JIC types C1 and C2. With regard to sex, there were no significant differences in any JHEQ scores between male and female patients who underwent osteotomy, whereas the total score in male patients undergoing THA was significantly higher than that in female patients (Tables 5 and 6).

**Table 4:** Post-operative JHEQ score at final follow-up

	Total		VAS		Pain		Movement	Mental
TRO	47.1 (4.2)		38.8 (7.5)		16.5 (1.5)		12.1 (2.2)	18.5 (1.8)
VO	51.4 (3.6)	*	30.8 (4.6)		18.0 (1.3)		15.5 (1.5)	17.2 (1.3)
THA	64.6 (2.8)	*	13.9 (3.0)	*	24.9 (0.7)	*	17.9 (1.4)	21.9 (1.8)

*JHEQ* Japanese Orthopaedic Association Hip Disease Evaluation Questionnaire, *TRO* transtrochanteric osteotomy, *VO* varus osteotomy, *THA* total hip arthroplasty

*p < 0.05

**Table 5:** JHEQ score in each pre-operative ONFH type and stage

	(Points)	TRO	VO	THA
Type C-1				
Total score		57.0 (5.3)	52.0 (3.8)	62.6 (3.9)
VAS		33..3 (3.3)	29.4 (5.0)	14.5 (4.7)
Pain score		20.0 (3.6)	18.2 (1.5)	24.8 (1.1)
Type C-2				
Total score		44.2 (4.9)	47.5 (12.1)	65.6 (4.0)*
VAS		43.0 (9.7)	40.0 (12.9)	14.2 (4.1)*
Pain score		15.5 (1.6)	16.0 (2.7)	24.7 (0.9)*
Stage 2				
Total score		52.6 (7.3)	54.6 (5.1)	57.2 (9.4)
VAS		22.0 (7.3)	20.6 (5.0)	28.0 (11.2)
Pain score		18.6 (2.6)	18.3 (1.9)	21.4 (2.8)
Stage 3A				
Total score		47.5 (5.9)	46.8 (4.9)	69 (4.0)*
VAS		45.0 (10.6)	45.0 (7.0)	10.8 (3.1)*
Pain score		16.7 (2.2)	17.5 (1.9)	26.7 (1.0)*

Japanese Orthopaedic Association Hip Disease Evaluation Questionnaire

*ONFH* osteonecrosis of femoral head

Results are expressed as mean (standard deviation), *p < 0.05

*TRO* transtrochanteric osteotomy, *VO* varus osteotomy, *THA* total hip arthroplasty

**Table 6:** JHEQ score in each post-operative ONFH type and gender

	(Points)	Total	VAS	Pain
Post-op				
Type B		57.2 (4.3)*	24.1 (5.0)*	20.5 (1.6)*
Type C-1		44.3 (3.6)*	40.8 (5.8)	14.9 (1.0)
Type C-2		37.5 (4.3)	55.0 (15.5)	13.5 (1.6)
Gender (osteotomy)				
Male		50.1 (3.7)	33.8 (5.0)	17.6 (1.3)
Female		50.1 (4.5)	33.6 (6.5)	17.5 (1.8)
Gender (THA)				
Male		71.6 (2.9)*	10.3 (4.0)	25.3 (0.8)
Female		59.4 (4.0)	16.7 (4.3)	24.5 (1.0)

Results are expressed as mean (standard deviation)

*post-op*. post-operative ONFH type

*p < 0.05

## Discussion

While managing ONFH using THA has generally resulted in good functional improvement and pain relief, joint-preserving surgeries are considered important because of poor long-term outcome after hip replacement in younger patients with ONFH [20–22]. Despite advancements in joint-preserving surgeries and the generally accepted principle of early intervention using the least damaging technique to preserve the femoral head, THA may still be required for patients with secondary OA. Inappropriate patient selection is one reason for poor outcomes after osteotomy [23, 24], and more efficient patient selection is mandatory to improve the selection of relevant treatment options. PROM metrics are important factors for evaluating the therapeutic effects of possible discrepancies between surgeons and patients with respect to patients’ postoperative satisfaction, pain relief, and ADL. Some authors have reported good short-term results in terms of patient satisfaction after several surgeries [25–27]. However, there is a paucity of large cohorts with osteotomies and THA comparisons for ONFH patients in terms of PROMS, which is useful for the decision-making process of whether to preserve the joint.

In this study, clinical evaluations of patients who underwent surgeries over ten years revealed that all surgeries generally resulted in good pain relief and functional improvement, and the total and pain scores of patients who underwent THA were significantly higher than those of patients who underwent TRO and CVO. Furthermore, the THA conversion rate of patients who underwent TRO was significantly higher than that of patients who underwent CVO, and the clinical evaluations of total and pain scores of the patients with TRO did not improve compared to the preoperative score. In addition, postoperative PROMS over ten years after the surgeries showed that the total and pain scores of patients with THA were significantly higher than those of patients with TRO and CVO, while the PROMS score did not change between patients with TRO and CVO. Several authors have reported that CVO is better than TRO in terms of operation time, amount of blood loss, postoperative collapse, osteoarthritic change, and postoperative survival [28–30]; our results also indicated that patients with CVO can achieve better clinical outcomes than those with TRO, although there is no difference in terms of postoperative PROMs and satisfaction. Recent advancement of cell therapy also can promote clinical outcomes [31], and the integration of osteotomy and cell therapy may have a potential to enhance clinical outcomes and patient’s satisfaction. From the point of view of cost-effectiveness, joint-preserving surgeries were reported to have the potential to be a highly cost-effective alternative if it leads to a delay in the need for THA of 5 years or longer [32]. However, based on current implant progress and performance, several authors mentioned that an earlier THA is cost-effective when considering an individual’s quality of life and life expectancy; it depends on how joint-preserving surgeries ensure the postoperative long-term good clinical outcomes and satisfaction [33].

In terms of surgical indications, patients with preoperative ONFH type C2 and stage 3A (already collapsed) who underwent THA showed better total and pain scores than those who underwent osteotomies which indicate that patients with preoperative type C2 and preoperatively collapsed ONFH may not have good postoperative satisfaction if they underwent osteotomies. Moreover, we have to make postoperative ONFH type B when performing osteotomy to achieve long-term patient satisfaction after surgery. As clinical outcomes after TRO and CVO are also reported to be associated with the preoperative stage and size of the ONFH and the postoperative intact ratio [34, 35], our study indicates that proper patient selection is needed for surgical osteotomy, especially in the TRO procedure. Furthermore, this study indicates that osteotomies should be performed in the early stages of the disease before the marked collapse of the femoral head, especially type C1 and stage 2 for the postoperative long-term good clinical outcomes and satisfaction after osteotomy. In patients who were not categorized as postoperative ONFH type B, even if they meet the indication for osteotomies with regard to the preoperative intact area, we may choose arthroplasty with a comprehensive patient explanation.

Our study has several limitations. First, the number of patients in this study was small because of the small proportion of patients who had undergone uncommon procedures of TRO and CVO at our institution, as in every institution. Therefore, we believe that the current results reflect the real outcomes of surgery but performing a multicentre study would be helpful to increase the power of the study in the future. Second, the examination of multiple osteonecrosis was not sufficient to permit analysis. Third, the investigation of bone marrow oedema of the femoral head using MRI is important from the point of view of pain evaluation; we could not evaluate it completely in this multicentre study.

## Conclusions

This study found that CVO and THA are clinically effective treatments for ONFH, with significant improvements compared with preoperative scores. However, THA was associated with significantly higher PROMs and pain scores than those of CVO and TRO in long-term follow-up. Our results also demonstrate that osteotomies should be performed in the early stages of the disease, especially in patients with JIC type C1 and stage 2 patients for the postoperative long-term good clinical outcomes and satisfaction. However, greater attention should be paid to treating patients who were not categorized as postoperative ONFH type B, even if they meet the indication for osteotomies with regard to the preoperative intact area; they may need subsequent conversion to THA.

## Data Availability

The data that support the findings of this study are not available due to ethical concerns.
